# Pointing Behavior in Infants Reflects the Communication Partner’s Attentional and Knowledge States: A Possible Case of Spontaneous Informing

**DOI:** 10.1371/journal.pone.0107579

**Published:** 2014-09-11

**Authors:** Xianwei Meng, Kazuhide Hashiya

**Affiliations:** 1 Graduate School of Human-Environment Studies, Kyushu University, Fukuoka, Japan; 2 Faculty of Human-Environment Studies, Kyushu University, Fukuoka, Japan; Birkbeck, University of London, United Kingdom

## Abstract

Inferring the epistemic states of others is considered to be an essential requirement for humans to communicate; however, the developmental trajectory of this ability is unclear. The aim of the current study was to determine developmental trends in this ability by using pointing behavior as a dependent measure. Infants aged 13 to 18 months (*n* = 32, 16 females) participated in the study. The experiment consisted of two phases. In the Shared Experience Phase, both the participant and the experimenter experienced (played with) an object, and the participant experienced a second object while the experimenter was absent. In the Pointing Phase, the participant was seated on his/her mother’s lap, facing the experimenter, and the same two objects from the Shared Experience Phase were presented side-by-side behind the experimenter. The participants’ spontaneous pointing was analyzed from video footage. While the analysis of the Shared Experience Phase suggested that there was no significant difference in the duration of the participants’ visual attention to the two objects, the participants pointed more frequently to the object that could be considered “new” for the experimenter (in Experiment 1). This selective pointing was not observed when the experimenter could be considered unfamiliar with both of the objects (in Experiment 2). These findings suggest that infants in this age group spontaneously point, presumably to inform about an object, reflecting the partner’s attentional and knowledge states.

## Introduction

Human communication is based on mutual “understanding” about others’ knowledge states as well as their attentional states. A communication participant has to continuously focus and adjust his/her verbal and nonverbal expressions based on the partner’s attentional and knowledge states, which are inferred through verbal and nonverbal expressions, and contextual information [1]–[5]. The developmental origin of this aspect of communication has drawn researchers’ attention, especially since the turn of this century. Studies have examined preverbal infants’ communicative expressions in various social contexts and clarified how such communication emerges and transforms over the course of development. For example, infants seem to be able to interpret other people’s communicative expressions, reflecting whether, and how, they have previously shared an experience with the communication partner regarding a particular object [6]–[9]. Furthermore, infants communicate in a way that is appropriate to the partner’s epistemic states [10], even in a false-belief type of situation [11].

These studies all show that preverbal infants effectively respond to communication initiated by others, including the monitoring of common elements that are shared with the individual. Moreover, these studies suggest that preverbal infants have some form of understanding regarding others’ goals and communicative intentions [12]. However, it is worth noting that most of the studies were primarily concerned with comprehensive aspects of infants’ communication. Considering the interactive process of communication, the development of spontaneous production has to be examined in order to understand the origin of human communication [13].

One major communicative behavior directly related to this topic is initiating joint attention, which occurs when the infant attempts to direct others’ attention towards the object of his/her attention through voluntary gaze shift and pointing [14]–[17]. In particular, the behavior of pointing has been extensively studied as one form of spontaneous communicative behaviors [18]–[20]. Classically, the communicative functions of pointing have been classified into the two major categories of *imperative* and *declarative,* which correspond to the motivation of the individual *to obtain something* and *to share interest with or inform others*, respectively [21], [22] (also see the discussion of an *interrogative* function of pointing [23]). Especially, declarative pointing in social-communicative contexts has been thought to reflect the understanding of others as mental agents, and the cooperative motives of sharing attention and interest with others [4], [22], though further examination regarding the possibility of “leaner” interpretations that do not include reading others’ minds (e.g., the infant might point just to ensure that the adults were engaged with the target, as an expression of his/her desire to enter into shared attentional states) is required [24]–[26].

Declarative pointing in response to events has been typically observed at around 12 months of age. Infants at this age have exhibited a higher tendency to point to an event when the adult is not attending to it, compared to when the adult is already attending to it [18], [27]. Moreover, the infants seemed to be most satisfied when others attended to their intended referent and shared interest in it [20], [28]. These findings imply that infants adapt their declarative pointing to the attentional states of others. However, it is also important to note that human communication not only requires the individual to track the communication partner’s attentional states, but also involves awareness of the other person’s knowledge states (or the conceptual ground that the partner shares with the individual) [4], [29], [30]. Since explicit signs representing the individual’s knowledge states are often unobservable, these states need to be estimated through an interactive context or based on previous experiences [31], [32]. In this estimation process, an important assumption is that the knowledge should have been shared with that particular partner through an experience. Though previous studies have suggested that 12-month-olds point more frequently to provide information to another person who is looking for an object without particular knowledge about the object’s location, as compared to situations in which he/she has knowledge of the object’s location [10], [33], this type of pointing could be interpreted as a response elicited by the experimenter’s particular actions embedded in the “searching” context, such as frowning, raising his/her hands, or asking, “Where has it gone?”.

Liebal et al. (2010) investigated whether infants’ previously shared experience with a person affects their pointing behavior at a later time. In their study, the infant first shared an experience involving one set of objects (Set 1) with one experimenter (E1), and then shared another experience with a different set of objects (Set 2) with another experimenter (E2). Then, the infant was moved to another room with either E1 or E2 and was shown two photographs: one of an object from Set 1 and the other from Set 2. The researchers found that by 18 months, the infants selectively pointed to the photographs that were relevant to the experience they had shared with that particular experimenter [13].

Thus, infants’ selective pointing that might reflect knowledge shared between the infant and another person has been observed, in the situation where joint attention has already been established. This type of pointing might reflect the infant’s motivation to share an attitude about the object with the other person, as Liszkowski et al. (2007a) have suggested in their “sharing hypothesis” [18]. From a social function view, sharing such an attitude with other group members has been regarded as serving to solidify group membership [4].

However, it is also worth noting that in the “sharing” setup in Liebal et al. (2010), no informative gap was expected between the infant and the experimenter with regard to the attentional states for the “new” object for the experimenter (i.e., from the perspective of the infant, “You find it when I do”), while a gap was expected in the knowledge states (“You do not know about it, but I do”). In such a situation, a difficulty has been suggested (even by the authors themselves) in separating the infants’ recognition of the partner’s epistemic states from their associative reasoning on the basis of observed sequences of actions [13]. To further our knowledge in this field, examining infants’ pointing in situations where there is also an expectation of a gap in attentional states (“You do not find it, but I do”) should be fruitful, as a false-belief situation has aided in separating explanations of behavior using Theory of Mind versus mere associationism, in accordance with the commentaries regarding Premack and Woodruff (1978) [34]–[37]. Moreover, systematic testing in this communicative context should further our understanding of pointing in infancy, especially with respect to infants’ understanding of the interaction between partners’ attentional and knowledge states.

Therefore, the current study focused on spontaneous pointing produced by infants at the age at which they are capable of tracking epistemic states, in situations in which the joint-attention frame regarding an object has not yet been established between the infant and another person, and in which no expressive cue that could elicit pointing from the other person is available. More specifically, in the current study, the infant participant interacted with the experimenter (E1) and one object in one condition and with the mother and another object in another condition. Thus, the infant was equally familiar with both the objects, whereas E1 was only familiar with one object. Following this phase, the infant faced E1 across a table, and the two objects were presented in windows behind E1. We examined whether, and how, infants’ spontaneous pointing could be selective in such a situation–one in which the joint-attention frame was obstructed. One might predict that selective pointing would be biased towards the object that the infant and E1 previously shared based on Liebal et al.’s (2010) findings. That is, this tendency might be robust enough to be independent of whether or not the joint-attention frame has formed [13].

An alternative possibility is that the infant’s selective pointing is biased toward the “new” object for E1. If this tendency were shown, it would suggest a spontaneous informing nature of pointing, possibly reflecting that the “new” object has relatively higher informative value to E1, compared to the “old” object [38]. In the current setup, E1 has no chance to acquire information about the “new” object without the infant’s explicit pointing. This particular setup might emphasize the informing function of pointing, rather than sharing emotions or attitudes [39]. In other words, the experimental setup of the previous study that led the infant to share his/her attentional states with the experimenter (“You find it when I do”) might have suppressed the informative nature of their pointing, since the “new” object for the experimenter is no longer new in this situation [13].

## Experiment 1

### Methods

#### Ethics Statement

All participants were recruited from a database of children whose parents had volunteered to participate in infant studies at Kyushu University. Written informed consent was obtained from the children’s caregivers before the experiment was conducted. The procedure was approved by the ethics committee of the Faculty of Human-Environment Studies at Kyushu University.

#### Participants

Sixteen 13- to 18-month-old infants (nine females; age *M* = 15 months, 1 day, *range* = 13 months, 6 days to 18 months, 9 days) participated in this study. Two other infants were excluded from the final sample due to fussiness (*n* = 1) and shyness (*n* = 1). To simplify the experimental situation [13], we designed the experiment to include the mother in the procedure. However, mothers were instructed to behave in a specific way during the Shared Experience Phase and not to initiate any interaction or communication with their infants during the Pointing Phase.

#### Materials and Set-up

An open booth with three draped walls was set up in a quiet room for the experiment. Both experimental phases were completed in this booth. The central wall had one red light in the center, and two openings (or windows, size: 25×25 cm, height: 110 cm) located 30 cm to the right and left of the red light. In the booth, the infants were seated on their mothers’ laps, 200 cm from the central wall and facing Experimenter 1 (E1), who sat across the table. Experimenter 2 (E2) assisted E1 and handled the objects (e.g., removing the objects from a 24×24×15 cm container) in the Shared Experience Phase, and controlled object presentation in the Pointing Phase.

Six pairs of objects (12 objects in total) were used in the experiment. The two objects in each pair were from the same category (e.g., two balls or two puppets), but differed in color and shape. Furthermore, each object made a different sound when it was played with in a particular way. The purpose of the sound was to attract the participants’ attention and to serve as a cue to help the infants memorize the objects.

**Figure 1:**
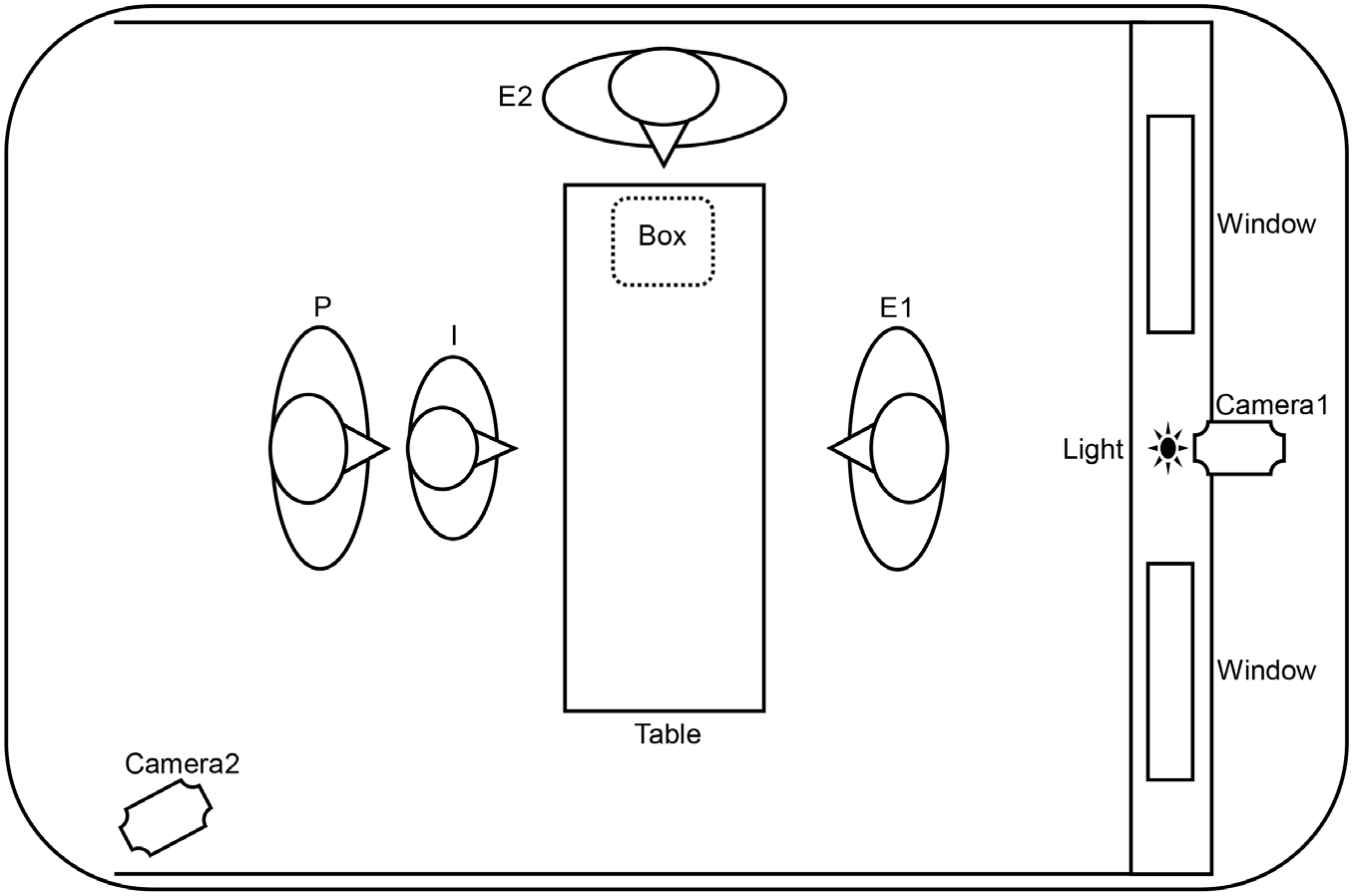
The experimental setup and materials.

Two cameras (SONY DCR-HC96) recorded all scenes from two different angles (Figure 1).

#### Procedure

Each infant completed a warm-up phase to establish a cooperative relationship with the experimenters. Infants, caregivers, E1, and E2 played with toys that were not used in the experiment in a corner of the same room as the experimental booth. The warm-up phase was terminated when the following conditions were met: (1) the infant displayed positive emotions towards the experimenters and interacted with them in a positive way; and (2) the infant gave the toys to the experimenters, both responsively and spontaneously.

Each trial of the main experiment consisted of two phases: “Shared Experience” and “Pointing.” The test continued until the infant grew obviously tired or fussy, or after the completion of six trials.

*Shared Experience Phase*. The Shared Experience Phase started upon completion of the warm-up phase, and after the infant, mother, and E1 took their positions in the booth. On each trial, infants experienced both *Shared-with-E1* and *Shared-with-Mother* conditions, as described below. One of the six object pairs was used in each trial.

In the *Shared-with-E1* condition, the infant and E1 played together with one object from the object pair across the table. Playing allowed E1 to become familiar with the particular object while interacting with the infant. The procedure for *Shared-with-E1* was as follows (on trials in which the *Shared-with-Mother* condition was conducted first, E1 entered the booth after the *Shared-with-Mother* condition):

1. E1 looked at the infant with a positive facial expression and talked to the infant using calm and positive vocal expressions in order to capture his/her attention (e.g., “Hello again! I am happy to meet you!”, in Japanese).
2. E2 entered the booth with a container in her arms. She sat down next to the table and hid the container under the table; therefore, the infant, mother, and E1 could not see what was inside it. E2 then opened the container, removed one of the objects, and silently passed it to E1 with no obvious facial expressions.
3. E1 showed the object to the infant and said, “Look! See what I have got! It’s very cute, isn’t it?” Then, E1 operated the object so it generated a sound to draw the infant’s attention towards the object.
4. E1 said to the infant, “Do you want to try? It is really fun!” and passed the object to the infant. E1 praised the infant when he/she played with the object, saying “Oh! You are very good at playing with it!”.
5. E2 covertly informed E1 when 60 s had elapsed. E1 terminated the interaction with the infant, and E2 put the object back into the container.

If the *Shared-with-Mother* condition followed the *Shared-with-E1* condition, E1 then left the booth, saying “Bye now! I’ll be back!”.

In the *Shared-with-Mother* condition, the procedure was identical to that of the *Shared-with-E1* condition except that the mother, not E1, shared an experience with the infant. The other object from the same pair as the *Shared-with-E1* condition was used (thus, the infant could infer that E1 was not familiar with that particular object).

The order of the *Shared-with-E1* and *Shared-with-Mother* conditions was counterbalanced across trials for each participant. The order of object-pair presentations, and which object in the pair was assigned to the *Shared-with-E1* condition were also counterbalanced.

*Pointing Phase*. E1 remained in the same position if the Shared Experience Phase ended with the *Shared-with-E1* condition; otherwise, E1 returned to the booth at this point.

1. E1 looked at the infant, displayed a positive facial expression, and talked to the infant using calm and positive vocal expressions in order to capture the infant’s attention (e.g., “Hello again! I am happy to meet you!”).
2. When the infant’s attention towards E1 was confirmed using an online video monitor, E2 (who was hiding behind the wall) flashed a red light behind E1 three times.
3. When the infant’s attention to the light was confirmed, E2 presented the same pair of objects that was used in the preceding Shared Experience Phase in the right and left windows.

The infants had experienced both objects in either the *Shared-with-E1* condition or the *Shared-with-Mother* condition. However, only one of the objects had been shared with E1; thus, the other object could be regarded as new for E1. The position of object presentation (left or right) was counterbalanced across trials.

The Pointing Phase ended when the infant pointed to one of the objects or after 60 seconds had elapsed. In response to the infant’s pointing, E1 turned her head towards the object that the infant had pointed to and used a mildly positive verbal expression, such as “Oh! There it is!” In this phase, E1 continued to communicate with the infant until the infant pointed to one of the objects in the windows, but not in an interrogative manner (i.e., E1 avoided asking the infant to tell her what was happening). The trial procedure was repeated up to six times. A trial lasted for approximately three minutes.

### Coding and Reliability

Each trial of the Shared Experience and Pointing Phases were coded separately so that the coders had no information about which object in the Pointing Phase had been used with the mother or E1 during the Shared Experience Phase. The following test-trial content was coded: (1) infants’ looking time towards the objects in the Shared Experience Phase (i.e., the duration in which the objects were obviously present in the infants’ observation range) (intra-observer reproducibility ICC_(1,2)_ = .98, *p*<.001; 50% of the data were coded twice to calculate reliability); (2) the object the infant pointed to first in the Pointing Phase (defined as when the infant extended his/her arm either fully or slightly bent and extended an index finger in the direction of the objects) (inter-observer agreement Cohen’s *κ* = .93, *p*<.001; 100% of the data were coded by two independent coders). If the object the infant pointed to could not be clearly discerned, the direction of the infant’s gaze was recorded instead.

### Results and Discussion

All infants produced at least one pointing action in an average of four trials in the Pointing Phase (*M* = 4, *SD* = 1.67, *range*_ trials_ = 1–6). Only the data from trials that included pointing in the Pointing Phase were analyzed.

**Figure 2:**
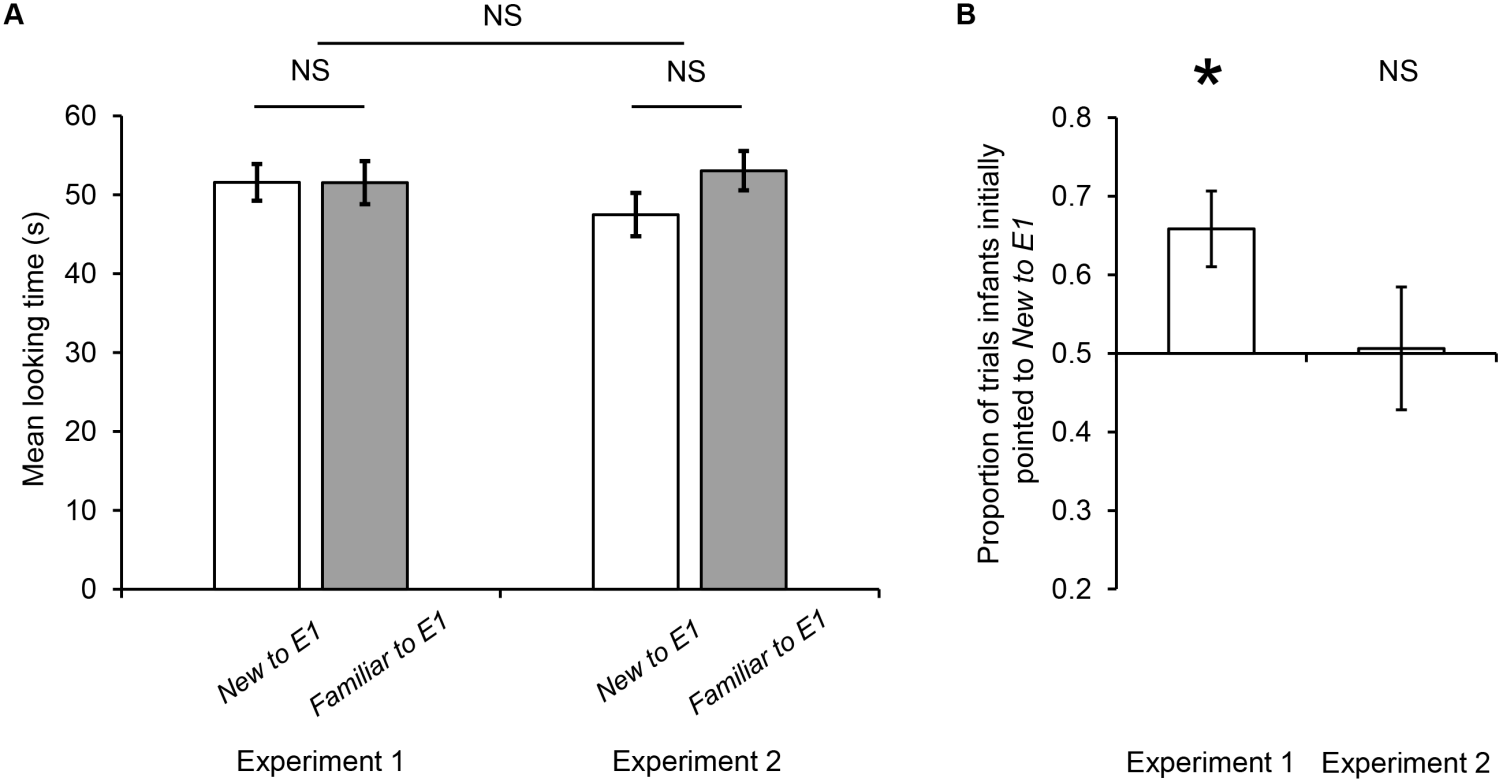
Test results from Experiments 1 and 2. (a) Mean looking time towards each object in the Shared Experience Phase in Experiments 1 and 2. (b) Proportion of trials in which infants initially pointed to the object that was “new” to E1 in the Pointing Phase of Experiments 1 and 2 (*p = .005). For both panels, error bars represent SEM.

Results showed no significant difference in looking time between the two objects (i.e., New to E1 and Familiar to E1) in the Shared Experience Phase (*M*_New to E1_ = 51.58, *SD* = 9.34; *M*_Familiar to E1_ = 51.55, *SD* = 10.9; *t*(15) = .018, *p* = .986, two-tailed, *r* = .001), indicating no significant difference in the visual experience with the two objects. To measure each infant’s pointing performance in the Pointing Phase, we counted the number of trials in which the infant initially pointed to each object and computed the proportion of *N*_New to E1_/(*N*_New to E1_+ *N*_Familiar to E1_) as an index. We found that infants initially pointed to the *New to E1* object on 66% of all the trials that included pointing on average, and a one-sample t-test showed that this was significantly above chance (50%) (*t*(15) = 3.29, *p* = .005, two-tailed, 95% CI = .56–.76, *d* = .869). These results demonstrate that despite the lack of difference in the visual experience in the Shared Experience Phase, in the Pointing Phase, infants had a bias to point to the object that they did not share with E1 (see Figure 2).

These results may suggest that the infants pointed to the object that E1 did not know, reflecting their estimation of E1’s knowledge states. However, there are several alternative explanations. For example, the infants might have pointed to the object based on a preference that had been formed during the shared experience with their mothers, or based on a desire to play with E1 and the “new” object together (i.e., imperative pointing; [21]). Alternatively, the infants may have pointed to the object that they experienced with their mother based on a desire to share attitudes/feelings with her (i.e., sharing pointing; [13]).

To evaluate the possibility of imperative pointing as an explanation of the current results, we conducted a post-hoc analysis to investigate the number of repeated pointing and reaching behaviors because these behaviors can be considered requesting behaviors [20]. If infants’ pointing reflects a request for the object, then we would expect them to repeat the pointing and reaching behaviors until they obtain the object. We analyzed occurrences of these behaviors in the duration between the onset of E1’s response to the infant’s pointing towards the object and the offset of the object presentation in the Pointing Phase, which was a period of approximately seven seconds. We found that infants’ repeated pointing and reaching occurred very rarely: out of the 64 trials with pointing, repeated pointing occurred on only four trials and reaching occurred on only eight. When we analyzed the data after excluding trials with repeated pointing or reaching, the results did not change (*p*<.001, *d* = 1.19) from those reported above.

Concerning the possible interpretation that infants pointed to the object based on their desire to share attitudes/feelings about that particular object with the mother, we investigated explicit communicative actions directed towards the mother within 10 s of pointing onset. Inspection of the videos indicated that such pointing was not observed on 61/64 trials, suggesting that infants’ pointing was not likely directed towards their mother for sharing attitudes/feelings.

These results partly support the idea that the infants’ pointing behavior that was observed in Experiment 1 was directed towards the experimenter, reflecting the shared experience. The following control experiment was conducted to provide more direct evidence.

## Experiment 2

In Experiment 2, we investigated whether the infants’ pointing behavior observed in Experiment 1 reflected the shared experience between the infants and the experimenter, but not the shared experience between the infant and the mother. In this experiment, in the Pointing Phase, a new experimenter (E3) who was not involved in the Shared Experience Phase of Experiment 1 and thus had no shared experience with the infant regarding either object, interacted with the infant. All other procedural details regarding the Shared Experience Phase were identical to those in Experiment 1. Therefore, if the results of Experiment 1 were driven by the shared experience between the infant and mother, we would expect that the results of Experiment 1 would be replicated. That is, the infants should point towards the objects that might be new for E1 more often than they point to the other object. However, if the results of Experiment 1 reflected the infant’s use of E1’s knowledge states (and not the mother’s), we would expect that the results of Experiment 2 would differ from those of Experiment 1.

### Methods

#### Participants

Another group of sixteen 13- to 17-month-old infants (seven female; age *M* = 15 months, 15 days, *range* = 13 months, 17 days to 17 months, 26 days) participated in this experiment. Six other infants were excluded from the final sample due to fussiness (*n* = 4) and shyness (*n* = 2). The infants’ mothers also participated in the experiment. However, the mothers were instructed to behave in a specific way during the Shared Experience Phase and not to initiate any interactions or communication with their infant during the Pointing Phase.

#### Procedure

Experiment 2 was identical to Experiment 1, except that E3 was absent during the Shared Experience Phase and interacted with the infants in the same manner as E1 did in the Pointing Phase of Experiment 1. E3 participated in the warm-up phase to establish a cooperative and collaborative relationship with the infants.

### Coding and Reliability

The same coding procedure as in Experiment 1 was used, and the following behaviors observed on the test trials were coded: (1) the time the infants spent looking towards the objects in the Shared Experience Phase (intra-observer reproducibility ICC_(1,2)_ = .99, *p*<.001; 50% of the data were coded twice to calculate reliability), and (2) the object that the infant pointed to first in the Pointing Phase (inter-observer agreement: Cohen’s κ = .97, *p*<.001; 100% of the data were coded).

### Results and Discussion

The results showed that, as in Experiment 1, there was no significant difference in the duration of looking towards the two objects in the Shared Experience Phase (*M*_New to E1_ = 47.5, *SD* = 11.02; *M*_Familiar to E1_ = 53.07, *SD* = 9.99; *t*(15) =  −1.619, *p* = .126, two-tailed, *r* = .272), indicating that there was no significant difference between the visual experiences with the two objects. Moreover, there was no significant difference in the infants’ total looking time towards the two objects in the Shared Experience Phase between Experiments 1 and 2 (*M*_Experiment 1_ = 51.57, *SD* = 9.71; *M*_Experiment 2_ = 50.29, *SD* = 7.96; *t*(29) = .408, *p* = .687, two-tailed, *d* = .149), suggesting that the infants in Experiment 1 and s performed similarly in the Shared Experience Phase.

Identical to Experiment 1, to measure each infant’s pointing behavior in the Pointing Phase, we counted the number of trials in which he/she initially pointed to each object, and computed the proportion of *N*_New to E1_/(*N*_New to E1_+ *N*_Familiar to E1_) as an index. However, in clear contrast to Experiment 1, the infants initially pointed to the *New to E1* on 51% of all the trials that included pointing on average, which was not significantly different from chance (50%) (*t*(15) = 0.08, *p* = .937, two-tailed, 95% CI = .34–.67, *d* = .02). Furthermore, there was no significant difference between the two experiments, with reference to the total number of trials with pointing in the Pointing Phase (Wilcoxon rank sum test, *p* = .626, two-tailed, *r* = .086). That is, as in Experiment 1, each infant in Experiment 2 produced at least one pointing response in an average of four trials (*M* = 3.94, *SD* = 1.53, *range* = 1–6). Therefore, there was no difference in the frequency of pointing in the Pointing Phase between the two experiments.

We also evaluated the possibility that the imperative interpretation could explain the current results by analyzing the number of repeated pointing and reaching behaviors in the duration between the onset of E1’s response to the infant’s pointing towards the object and the offset of object presentations in the Pointing Phase, which lasted for approximately seven seconds. Similar to that observed in the previous experiment, repeated pointing and reaching behaviors occurred very rarely (across the 63 trials in which pointing was observed, repeated pointing occurred on three trials and reaching occurred on six). Further, when we excluded the trials in which repeated pointing or reaching behavior was observed, the findings did not change (*p* = .98, *d* = .007).

The results of Experiment 2 suggest that when objects that the infants had not shared with the experimenter appeared behind the experimenter, selective pointing was not observed. The combined results of the two experiments indicate that infants point selectively only when the experimenter might be considered familiar with one of the objects (as in Experiment 1). Thus, the difference in pointing behavior observed in Experiment 1 most plausibly reflects a shared experience between the infant and E1, and not with the mother.

## General Discussion

The current study demonstrated that infants in the first half of their second year had a spontaneous tendency to selectively point to an object that the communication partner might not know, reflecting a previously shared experience in a context that lacked a joint-attention frame on the objects’ appearance. Experimental studies on spontaneous production of pointing have suggested that infants might point for others, with an understanding of their epistemic states [13] [18]. In a departure from previous findings, the current study systematically examined infants’ pointing behavior in a situation in which the joint-attention frame had not been established. The results demonstrated that, when the infant had experienced two objects (thus both were “old”) but one object could be thought of as “new” to the communication partner, the infant spontaneously pointed to the latter object when both objects were presented behind the partner.

One goal for participants in a communicative situation should be a reduction in the gap in attentional and knowledge states between oneself and the communication partner. This can be achieved in two ways: by transforming one’s state (through learning processes), and by helping the partner to transform his/her state. Providing information should be one major process for achieving the latter. The pointing behavior observed in the current study might be viewed as a form of informing directed towards the experimenter in the sense that the infant pointed to the object that the communication partner might not know. However, considering previous studies, it is possible that the pointing behavior observed in the current study served other functions, albeit not in a mutually exclusive manner. Therefore, to clarify the position of the current results, we discuss the following interpretations of pointing behavior and contrast them with ours: *imperative pointing*, *pointing for sharing*, and *interrogative pointing*.

### Imperative Pointing

Pointing may serve an imperative function [21]. The infants in the current study may have been expressing the desire to obtain a particular object, based on a preference that had been formed during the shared experience with the mother or on the desire to play with the experimenter with the “new” object. However, the infants’ pointing behavior differed between the two experimental conditions. In other words, the infants changed their pointing strategy to reflect whether they had a shared experience regarding the object with the current communication partner. This clearly suggests that the pointing behavior observed here was not solely an attempt to obtain the object that was involved in the shared experience with the mother. Moreover, in the Pointing Phase of the current study, even though the experimenter did not retrieve the object for the infant in response to a point, signs that the infants were making requests or expressing dissatisfaction were observed at a very low frequency. Specifically, the infants displayed repeated pointing and reaching behavior in only 6% and 12.5% of all trials with pointing behavior in Experiment 1 and in 5% and 10% of all trials with pointing behavior in Experiment 2, respectively. Thus, the infant’s pointing behavior in the current study seems to lack the typical behaviors that accompany object requests, indicating that the interpretation of pointing behavior as an expression of the motive of “I want that object!” is inadequate [20], [33], [40].

### Pointing for Sharing

It is also possible for infants to use pointing as a way of sharing attitudes towards a particular object [18], [22] (see also [24], [25]). Moreover, infants might point to an object to share attitudes with a particular adult based on a previously shared experience [13]. That is, the selective pointing observed in Experiment 1 might have been based on the sharing of attitudes with the mother about the objects they previously shared. However, explicit communicative actions towards the mother were rarely observed in the current experiments; more importantly, selective pointing was not observed in the control experiment (Experiment 2). These results suggest that the pointing behavior observed in the current study was not a reflection of a desire to share attitudes with the mother; however, the interpretation that the pointing behavior was a method of sharing with the experimenter (rather than the mother) remains compatible with the current results.

### Interrogative Pointing

Begus and Southgate [23] suggested that infants point in order to obtain information from others. In the current study, however, the infants had a similar degree of experience with the two objects in the Shared Experience Phase. Thus, it may be presumed that the infants had a similar level of knowledge about both. Moreover, when the joint-attention frame has been established, infants tend to point to the “old” object [13]. Consequently, the interrogative account does not seem to predict the selective pointing that was observed in Experiment 1.

At least in the context of the current study, infants seem to reliably point to an object that can be considered “new” and invisible for the communication partner. Considering the contrast with previous findings by Liebal et al. (2010), it would be rather surprising that infants at this age point flexibly, thereby reflecting the combination of different attentional and knowledge states [13]. The current results are in agreement with suggestions that infants have some level of understanding about the discrepancies in current attentional [41]–[43] and knowledge states [7]–[9] between themselves and their communication partners. Furthermore, they also demonstrate infants’ prosocial motives for interacting with others [44], [45], even in situations in which the experimenters do not show any explicit expression about such states [10], [33].

One might argue that the leaner explanation is applicable to the current data: the infants might have learned through previous experiences that it is more rewarding to lead the partner into an association with a previously unassociated object. However, previous studies have indicated that even when 12-month-olds point, they are not satisfied unless the adult shares their attention and interest in the object [20], [28]. Moreover, one cannot simply say that infants point to unassociated objects because they have learned that doing so is more rewarding, since this would not explain the finding that infants selectively point to “old” objects [13], implying a difference in the role of pointing depending on whether joint attention exists.

We cannot completely clarify what communicative motive drives infants to inform others about potentially new information. Infants might expect feedback from others after they provide the new information, or they might expect the partner to express surprise or pleasure when the new objects are seen. Infants might also have more self-based motives that do not involve a complex reference process. For example, they might automatically “embody” others in particular situations via empathic pathways [46]–[48] and then demonstrate their own novelty preferences from past experience. These hypotheses regarding underlying mechanisms should be tested in future works.

The experimental setup we prepared here did not allow for the object to be involved in a joint-attention frame, and thus might broaden the gap in information values of the object between the infant and the experimenter in the communicative context [38]. E1 has no chance to acquire information about the “new” object without the infant’s explicit pointing. This particular setup might have served to emphasize the informing function of pointing, rather than sharing emotions or attitudes (“You do not find the object that you do not know about, but I find it and I know about it”) [39]. Such a way of informing might be viewed as one essential part of cooperative behavior that enables transmission of information and provides the partner a chance to learn [49]–[51].

Recent studies have implied that preverbal infants seem to be prepared to receive culturally relevant knowledge from benevolent adults, based on specialized cognitive mechanisms adapted for social interaction, such as sensitivity to ostensive signals (e.g., direct eye contact [52]–[56]) or referential expectation (e.g., infants expect that ostensive signals will be followed by referential signals [57], [58]). Csibra and Gergely (2009) further argued that these specific cognitive mechanisms might enable the communication system called *natural pedagogy*, which is specifically adapted to allow the transmission of generic knowledge between individuals. However, as they noted, these studies have focused on the receptive side of cultural transmission. In other words, they have clarified that infants are astonishingly effective “learners” [59], [60].

The current results might provide new evidence that the preverbal infant is not only an effective learner, but is also a flexible participant in communication, and may also serve as an effective informant. This view might be properly supported by the theoretical framework of indirect reciprocity [61], which suggests that particular behavior towards the other potentially raises the actor’s adaptive fitness through the pathway of reputation among social group members [62], and opens the possibility of rational understanding about the evolutionary background of altruistic behavior. The infants’ spontaneous tendency to inform, a form of altruistic behavior [63], might be understandable based on the assumption that indirect reciprocity is rooted from early childhood in humans [64], [65]. Although the distance between the behavior of informing about an object’s appearance or location and the more formulated and culturally shaped form of teaching and education cannot be underestimated [50], [66], the view of the infant as an “effective informant” might provide a perspective worth consideration when attempting to further our understanding of the basis of human information transmission through communication.

## References

[1] Clark HH, Marshall CR (1981) Definite reference and mutual knowledge. In: Joshi AK, Webber BL, Sag, IA, editors. Elements of discourse understanding. Cambridge: Cambridge University Press. 10–63.

[2] Sperber D, Wilson D (1986a) Relevance: Communication and cognition. Oxford: Blackwell. 15–21.

[3] Sperber D, Wilson D (1987a) Precis of relevance: Communication and cognition. Behav Brain Sci 10: 697–754.

[4] Tomasello M (2008) Origins of human communication. Cambridge: MIT Press. 57–241.

[5] Murakami T, Hashiya K (2014) Development of reference assignment in children: A direct comparison to the performance of cognitive shift. Front Psychol. 5: 523. 10.3389/fpsyg.2014.00523 PMC4038857 24910629

[6] Tomasello M, Haberl K (2003) Understanding attention: 12- and 18-month-olds know what is new for other persons. Dev Psychol 39: 906–912. 12952402 10.1037/0012-1649.39.5.906

[7] Saylor MM, Ganea P (2007) Infants interpret ambiguous requests for absent objects. Dev Psychol 43: 696–704. 17484581 10.1037/0012-1649.43.3.696

[8] Liebal K, Behne T, Carpenter M, Tomasello M (2009) Infants use shared experience to interpret pointing gestures. Dev Sci 12: 264–271. 19143799 10.1111/j.1467-7687.2008.00758.x

[9] Moll H, Carpenter M, Tomasello M (2007) Fourteen-month-olds know what others experience only in joint engagement. Dev Sci 10: 826–835. 17973799 10.1111/j.1467-7687.2007.00615.x

[10] Liszkowski U, Carpenter M, Tomasello M (2008) Twelve-month-olds communicate helpfully and appropriately for knowledgeable and ignorant partners. Cogn 108: 732–739. 10.1016/j.cognition.2008.06.013 18721918

[11] Southgate V, Chevallier C, Csibra G (2010) Seventeen-month-olds appeal to false beliefs to interpret others’ referential communication. Dev Sci 13: 907–912. 20977561 10.1111/j.1467-7687.2009.00946.x

[12] Behne T, Carpenter M, Call J, Tomasello M (2005) Unwilling versus unable: Infants’ understanding of intentional action. Dev Psycholo 41: 328–337. 10.1037/0012-1649.41.2.328 15769189

[13] Liebal K, Carpenter M, Tomasello M (2010) Infants’ use of shared experience in declarative pointing. Infancy 15: 545–556. 10.1111/j.1532-7078.2009.00028.x 32693511

[14] Mundy P, Fox N, Card J (2003) EEG coherence, joint attention and language development in the second year. Dev Sci 6: 48–54.

[15] Mundy P, Willoughby J (1996) Nonverbal communication, joint attention, and early socioemotional development. In Lewis M, Sullivan MW, editors. Emotional development in atypical children. Hillsdale: Lawrence Erlbaum Associates. 65–87.

[16] Seibert JM, Hogan AE, Mundy PC (1982) Assessing interactional competencies: The early social-communication scales. Infant Ment Health J 3: 244–258.

[17] Kuroki M (2007) The effect of positive emotion on infants’ gaze shift. Infant Behav Dev 30: 606–614. 17416421 10.1016/j.infbeh.2007.03.008

[18] Liszkowski U, Carpenter M, Tomasello M (2007a) Pointing out new news, old news, and absent referents at 12 months of age. Dev Sci 10: F1–F7. 17286836 10.1111/j.1467-7687.2006.00552.x

[19] Bates E (1979) The emergence of symbols: Cognition and communication in infancy. New York: Academic Press. 69–140.

[20] Liszkowski U, Carpenter M, Henning A, Striano T, Tomasello M (2004) Twelve-month-olds point to share attention and interest. Dev Sci 7: 297–307. 15595371 10.1111/j.1467-7687.2004.00349.x

[21] Bates E, Camaioni L, Volterra V (1975) The acquisition of performatives prior to speech. Merrill Palmer Q 21: 205–224.

[22] Tomasello M, Carpenter M, Liszkowski U (2007) A new look at infant pointing. Child Dev 78: 705–722. 17516997 10.1111/j.1467-8624.2007.01025.x

[23] Begus K, Southgate V (2012) Infant pointing serves an interrogative function. Dev Sci 15: 611–617. 22925509 10.1111/j.1467-7687.2012.01160.x

[24] D’Entremont B, Seamans E (2007) Do infants need social cognition to act socially? An alternative look at infant pointing. Child Dev 78: 723–728. 17516998 10.1111/j.1467-8624.2007.01026.x

[25] Southgate V, van Maanen C, Csibra G (2007) Infant pointing: Communication to cooperate or communication to learn? Child Dev 78 (3): 735–740. 10.1111/j.1467-8624.2007.01028.x 17517000

[26] Doherty MJ, Anderson JR (1999) A new look at gaze: Preschool children’s understanding of eye-direction. Cogn Dev 14: 549–571.

[27] Legerstee M, Barillas Y (2003) Sharing attention and pointing to objects at 12 months: Is the intentional stance implied? Cogn Dev 18: 91–110.

[28] Liszkowski U, Carpenter M, Tomasello M (2007b) Reference and attitude in infant pointing. J Child Lang 33: 1–20. 10.1017/s0305000906007689 17340936

[29] Bruner J (1983) Child’s talk: Learning to use language. New York: Norton.

[30] Lee BPH (2001) Mutual knowledge, background knowledge and shared beliefs: Their roles in establishing common ground. J Pragmat 33: 21–44.

[31] Lucas AJ, Lewis C (2010) Should we trust experiments on trust? Hum Dev 53: 167–172.

[32] Harris PL, Koenig MA (2006) Trust in testimony: How children learn about science and religion. Child Dev 77: 505–524. 16686784 10.1111/j.1467-8624.2006.00886.x

[33] Liszkowski U, Carpenter M, Striano T, Tomasello M (2006) 12- and 18-month-olds point to provide information for others. Cogn Dev 7: 173–187.

[34] Bennett J (1978) Some remarks about concepts. Behavioral and Brain Sciences 1 (4): 557–560.

[35] Dennett DC (1978) Beliefs about beliefs [P&W, SR&B]. Behavioral and Brain Sciences 1 (4): 568–570.

[36] Harman G (1978) Studying the chimpanzee’s theory of mind. Behavioral and Brain Sciences 1 (4): 576–577.

[37] Premack D, Woodruff G (1978) Does the chimpanzee have a theory of mind? Behavioral and Brain Sciences 1 (4): 515–526.

[38] Shannon CE (1948) A mathematical theory of communication. Bell System Tech J 27: 379–423, 623–564.

[39] Fogarty L, Strimling P, Laland KN (2011) The evolution of teaching. Evol 65: 2760–2770. 10.1111/j.1558-5646.2011.01370.x 21967419

[40] Franco F, Butterworth G (1996) Pointing and social awareness: Declaring and requesting in the second year. J Child Lang 23: 307–336. 8936689 10.1017/s0305000900008813

[41] Butterworth G, Jarrett N (1991) What minds have in common is space: Spatial mechanisms serving joint visual attention in infancy. Br J Dev Psychol 9: 55–72.

[42] Caron A, Butler S, Brooks R (2002) Gaze following at 12 and 14 months: Do the eyes matter? Br J Dev Psychol 20: 225–239.

[43] Brooks R, Meltzoff AN (2002) The importance of eyes: How infants interpret adult looking behavior. Dev Psychol 38: 958–966. 12428707 10.1037//0012-1649.38.6.958 PMC1351351

[44] Kuhlmeier V, Wynn K, Bloom P (2003) Attribution of dispositional states by 12-month-olds. Psychol Sci 14: 402–408. 12930468 10.1111/1467-9280.01454

[45] Warneken F, Tomasello M (2007) Helping and cooperation at 14 months of age. Infancy 11: 271–294. 10.1111/j.1532-7078.2007.tb00227.x 33412734

[46] Iacoboni M, Woods RP, Brass M, Bekkering H, Mazziotta JC, Rizzolatti G (1999) Cortical mechanisms of human imitation. Sci 286: 2526–2528. 10.1126/science.286.5449.2526 10617472

[47] Gallese V, Rochat M, Cossu G, Sinigaglia C (2009) Motor cognition and its role in the phylogeny and ontogeny of action understanding. Dev Psychol 45: 103–113. 19209994 10.1037/a0014436

[48] Molnar-Szakacs I (2011) From actions to empathy and morality–a neural perspective. J Econ Behav Organ 77: 76–85.

[49] Alexander RD (1974) The evolution of social behavior. Annu Rev Ecol Evol Syst 5: 325–383.

[50] Caro TM, Hauser MD (1992) Is there teaching in nonhuman animals? Q Rev Biol 67: 151–174. 1635977 10.1086/417553

[51] Thornton A, Raihani NJ (2008) The evolution of teaching. Anim Behav 75: 1823–1836.

[52] Kobayashi H, Kohshima S (1997) Unique morphology of the human eye. Nat 387: 767–768. 10.1038/42842 9194557

[53] Kobayashi H, Hashiya K (2011) The gaze that grooms: Contribution of social factors to the evolution of primate eye morphology. Evol Hum Beh 32 (3): 157–165.

[54] Farroni T, Csibra G, Simion F, Johnson MH (2002) Eye contact detection in humans from birth. Proc Natl Acad Sci USA 99: 9602–9605. 12082186 10.1073/pnas.152159999 PMC123187

[55] Farroni T, Johnson MH, Menon E, Zulian L, Faraguna D, Csibra G (2005) Newborns’ preference for face-relevant stimuli: Effects of contrast polarity. Proc Natl Acad Sci USA 102: 17245–17250. 16284255 10.1073/pnas.0502205102 PMC1287965

[56] Senju A, Johnson MH (2009) The eye contact effect: mechanisms and development. Trends Cogn Sci 13 (3): 127–134. 10.1016/j.tics.2008.11.009 19217822

[57] Csibra G, Volein A (2008) Infants can infer the presence of hidden objects from referential gaze information. Br J Dev Psychol 26: 1–11.

[58] Moll H, Tomasello M (2004) 12- and 18-month-old infants follow gaze to spaces behind barriers. Dev Sci 7: F1–F9. 15323111 10.1111/j.1467-7687.2004.00315.x

[59] Csibra G, Gergely G (2009) Natural pedagogy. Trends Cogn Sci 13 (4): 148–153. 10.1016/j.tics.2009.01.005 19285912

[60] Csibra G, Gergely G (2011) Natural pedagogy as evolutionary adaptation. Phil Trans R Soc B 366: 1149–1157. 21357237 10.1098/rstb.2010.0319 PMC3049090

[61] Nowak MA, Sigmund K (1998) Evolution of indirect reciprocity by image scoring. Nat 393: 573–577. 10.1038/31225 9634232

[62] Mifune N, Hashimoto H, Yamagishi T (2010) Altruism toward in-group members as a reputation mechanism. Evol Hum Beh 31: 109–117.

[63] Warneken F, Tomasello M (2009) The roots of human altruism. Brit J Psychol 100: 455–471. 19063815 10.1348/000712608X379061

[64] Kato-Shimizu M, Onishi K, Kanazawa T, Hinobayashi T (2013) Preschool children’s behavioral tendency toward social indirect reciprocity. PLoS One 8: e70915. 23951040 10.1371/journal.pone.0070915 PMC3737253

[65] Meristo M, Surian L (2013) Do infants detect indirect reciprocity? Cogn 129: 102–113. 10.1016/j.cognition.2013.06.006 23887149

[66] Ando J (2012) On “Homo educans” hypothesis. In: Watanabe S, editor. CARLS series of advanced study of logic and sensibility. Tokyo: Keio University Press. 147–156.

